# Accuracy Assessment of Molded, Patient-Specific Polymethylmethacrylate Craniofacial Implants Compared to Their 3D Printed Originals

**DOI:** 10.3390/jcm9030832

**Published:** 2020-03-19

**Authors:** Dave Chamo, Bilal Msallem, Neha Sharma, Soheila Aghlmandi, Christoph Kunz, Florian M. Thieringer

**Affiliations:** 1 Department of Oral and Cranio-Maxillofacial Surgery, University Hospital Basel, CH-4031 Basel, Switzerland; dave.chamo@stud.unibas.ch (D.C.); neha.sharma@usb.ch (N.S.); christoph.kunz@usb.ch (C.K.); ft@swiss-mam.ch (F.M.T.); 2 Medical Additive Manufacturing Research Group (Swiss MAM), Department of Biomedical Engineering, University of Basel, CH-4123 Allschwil, Switzerland; 3 Basel Institute for Clinical Epidemiology and Biostatistics, Department of Clinical Research, University Hospital Basel, University of Basel, CH-4031 Basel, Switzerland; soheila.aghlmandi@usb.ch

**Keywords:** 3D printing, accuracy, additive manufacturing, craniofacial reconstruction, cranioplasty, molding, patient-specific implant, PMMA

## Abstract

The use of patient-specific implants (PSIs) in craniofacial surgery is often limited due to a lack of expertise and/or production costs. Therefore, a simple and cost-efficient template-based fabrication workflow has been developed to overcome these disadvantages. The aim of this study is to assess the accuracy of PSIs made from their original templates. For a representative cranial defect (CRD) and a temporo-orbital defect (TOD), ten PSIs were made from polymethylmethacrylate (PMMA) using computer-aided design (CAD) and three-dimensional (3D) printing technology. These customized implants were measured and compared with their original 3D printed templates. The implants for the CRD revealed a root mean square (RMS) value ranging from 1.128 to 0.469 mm with a median RMS (Q1 to Q3) of 0.574 (0.528 to 0.701) mm. Those for the TOD revealed an RMS value ranging from 1.079 to 0.630 mm with a median RMS (Q1 to Q3) of 0.843 (0.635 to 0.943) mm. This study demonstrates that a highly precise duplication of PSIs can be achieved using this template-molding workflow. Thus, virtually planned implants can be accurately transferred into haptic PSIs. This workflow appears to offer a sophisticated solution for craniofacial reconstruction and continues to prove itself in daily clinical practice.

## 1. Introduction

Severe head trauma, oncological resections, and congenital skull anomalies can lead to extensive cranial deformities [1,2]. A cranial defect (CRD) may cause functional, psychological, and esthetic problems for patients, making reconstruction necessary. Cranioplasty, the surgical repair of a congenital or acquired CRD, is a widely practiced procedure that not only aims to restore both the structure and function of the missing cranial bone but can also improve a patient’s neurological status [3,4].

Autologous bone grafts are regarded as the gold standard in the repair of any type of bone defect [5,6]. However, indications can be limited for cranial reconstruction due to the risk of infection, graft resorption, defect size, poor cosmetic outcome, and donor site morbidity [7]. The use of synthetic or alloplastic materials, such as alloys (Titanium [Ti]), ceramics (hydroxyapatite [HA]), acrylics (polymethylmethacrylate [PMMA]), or polymers (polyetheretherketone [PEEK]), has become increasingly popular. These alloplastic materials are now used regularly to reconstruct CRDs in a sufficiently cosmetic manner [6,8].

PMMA is the most frequently used alloplastic material for cranial reconstructions worldwide and has been considered the gold standard among neurosurgeons since the Second World War [9]. With long-term results, it has the advantage of being cost-effective and light weight without being thermoconductive [10]. It is also resistant to functional stress [11]. However, in complex craniectomy cases where a patient-specific Implant (PSI) is required, intraoperative molding of PMMA is still considered a challenging task. Other problems encountered with PMMA include the excessive heat generated by the exothermic reaction that occurs during the molding process, which might harm the surrounding tissues, or allergic reactions to monomers. In addition, the freehand PMMA molding technique is associated with an increased surgical time and often results in unacceptable cosmetic outcomes. Hence, to mitigate the problems associated with freehand implant fabrication, preoperatively or intraoperatively (extracorporeal) fabricated PMMA PSIs are used [12].

Recent digital innovations in computer-aided technologies have led to rapid development in personalized healthcare. With the integration of computer-aided design (CAD) and additive manufacturing, the exact size and shape of the CRDs are determined from the imaging data and transferred into a planning software to produce a PSI. This assimilation of technologies aids in patient-specific reconstructions using three-dimensional (3D) printed templates for the fabrication of customized prefabricated PMMA cranial implants [13]. The use of different reconstruction techniques has been reported in several studies, such as including a plastic bag with a wire mesh, a silicone mold from a 3D printed template, a one-part 3D printed mold, and a 3D printed mold with several parts [1,12,14,15,16]. Although the benefits of customized fabricated PMMA implants have been clearly described, few studies have evaluated the accuracy of PSIs and have only examined those fabricated using another workflow. Moreover, little is known about the reusability of silicone molds in the case of a revision operation.

Hence, the aim of this study is to evaluate the accuracy of molded PMMA PSIs in comparison to their virtually designed 3D printed template counterparts, which bridge the gap between molding and 3D printing.

## 2. Materials and Methods

### 2.1. Data Acquisition and Design of PSIs

**Figure 1:**
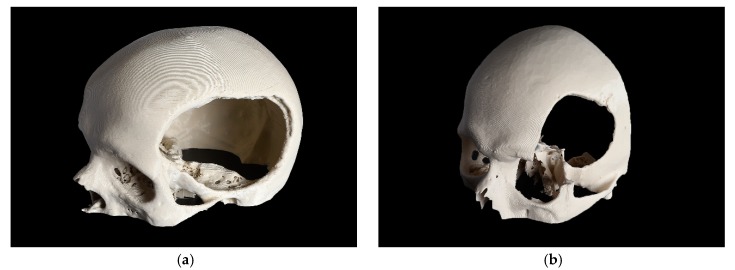
3D printed skull models with the defect area shown. (**a**) cranial defect (CRD); (**b**) temporo-orbital defect (TOD).

Based on the complexity of the craniofacial defect, two cases were selected and categorized as a CRD and a temporo-orbital defect (TOD), as shown in Figure 1.

The models’ high-resolution computed tomography (CT) data were acquired and consisted of the following parameters: matrix of 512 × 512 pixels; slice thickness of 1 mm; seed per rotation of 1 mm; gantry tilt of 0° and bone window setting. The Digital Imaging and Communications in Medicine (DICOM) data of the two-dimensional (2D) image slices from the CT scans were imported into the Materialise Interactive Medical Image Control System (MIMICS) medical software (MIMICS Innovation Suite v. 20.0, Materialise, Leuven, Belgium). Following this, threshold selection was performed, in which the inbuilt greyscales were selected using bone-specific Hounsfield units (HU) to mark the skull region. Using a semiautomatic segmentation tool in the software, a 3D volumetric image of the patient’s skull anatomy was generated. For a precise reconstruction of the virtual skull defect, established and proven computer reconstruction techniques, such as the mirroring function, were applied to replicate the corresponding contralateral intact anatomical skull region. A Boolean subtraction of the mirrored and defective portion of the skull was applied to generate the overall shape of the PSIs. The virtually planned PSIs’ files were smoothed and adapted with minor adjustments and were ultimately exported in the Standard Tessellation Language (STL) format.

### 2.2. 3D Printing and Scanning Protocol for PSI Templates

Subsequently, the STL files of the virtually planned templates were imported into the slicing software of a 3D printer (MakerBot Print v. 4.5.0.1729, MakerBot Industries, Brooklyn, New York, NY, USA). The software was set to the standard print settings with a layer height of 0.2 mm and an infill of 10 %. This ensures a relatively fast printing time, as the mechanical properties are not relevant for a template and only the accuracy counts. The templates were then printed in polylactic acid (PLA) precision filament (MakerBot PLA Filament (true white), MakerBot Industries, Brooklyn, New York, NY, USA) using a fused filament fabrication (FFF) desktop 3D printer (MakerBot Replicator+, MakerBot Industries, Brooklyn, New York, NY, USA). For optimal printing, support structures were generated in the slicing software of the 3D printer and were removed manually after printing. Postprocessing of the templates was done to adjust any small irregularities.

The 3D printed templates fabricated from PLA were digitized using a desktop optical 3D scanner (EinScan-SP, SHINING 3D Tech. Co., Ltd., Hangzhou, China). According to the manufacturer’s specifications, the scanner has a point distance of 0.17–0.2 mm and a camera resolution of 1.31 megapixels. The scanner uses structured white-light scanning technology and has a single shot accuracy of ≤ 0.05 mm. To acquire a 360° overview of the templates, the templates were fixed in two different positions, vertical and horizontal. The point cloud data generated by scanning the 3D printed templates were then converted and exported in STL format. 

### 2.3. Fabrication Process of the Silicone Mold and Subsequently of the PMMA PSIs

To reproduce the shape of the 3D printed templates, silicone molds were manufactured using an additive silicone material (Dublisil 30, Dreve Dentamid GmbH, Unna, Germany). For this purpose, a container with appropriate dimensions was used, which matched the size of the template. The container was half-filled with silicone and the template was positioned in the middle after being coated with paraffin to facilitate later removal. Contact of the PSI with the walls of the container should be avoided. After the first half of the silicone mold was cured, retention grooves were made in the mold. These grooves facilitated later positioning during PSI fabrication. The top half of the cured silicone mold can now be coated with paraffin and the rest of the container filled with silicone. After the silicone is completely cured, the template can be removed. The negative two-part molds were then sterilized in an autoclave using steam sterilization.

In this study, twenty PMMA PSIs were fabricated with these molds (*n* = 10 for each defect shape) using a high viscosity bone cement (PALACOS R, Heraues Kulzer GmbH, Hanau, Germany). Two experienced surgeons mixed the monomer liquid with the polymer powder according to the manufacturer’s instructions and individually fabricated the PMMA PSIs for each case [17]. First, the two molds were coated with paraffin and one half was placed on a stable base. After mixing the bone cement, placing it in the mold and covering it with the other half, the two halves were pressed and held under constant pressure until the exothermic curing process was finished. Post-curing, the PMMA PSIs were removed from the silicone molds and finished by trimming the surplus material. This workflow was repeated ten times for the CRD-PSIs and TOD-PSIs.

### 2.4. Scanning Process of the PMMA PSIs

The PMMA PSIs were digitized using cone-beam computed tomography (CBCT) (CS 9300, Carestream Dental LLC, Atlanta, GA, USA). Based on the system manufacturer’s recommendations, the optimal data acquisition parameters were selected (Table 1). A non-radiopaque holder was used to place the PMMA PSIs in the focus field during the scanning protocol.

The scan-volume data for the TOD-PSIs were smaller in size and, therefore, had a higher resolution than the scan-volume data for the CRD-PSIs. The segmentation and surface extraction of the CBCT DICOM data of the PMMA PSIs were then performed using the MIMICS medical software (MIMICS Innovation Suite v. 20.0, Materialise, Leuven, Belgium). Finally, the data from each digitized PMMA PSI were exported in the STL format.

### 2.5. Accuracy Analyses

**Figure 2:**
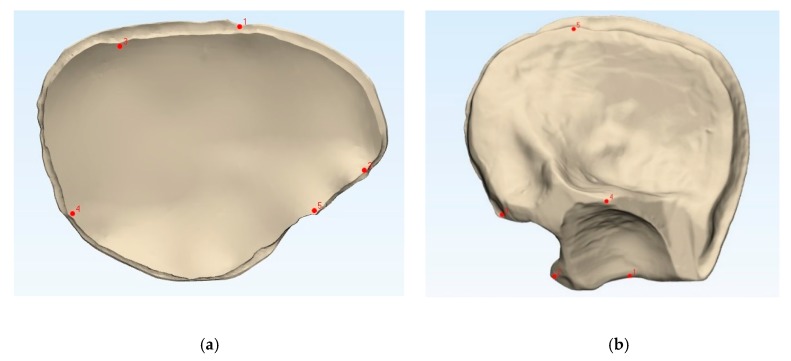

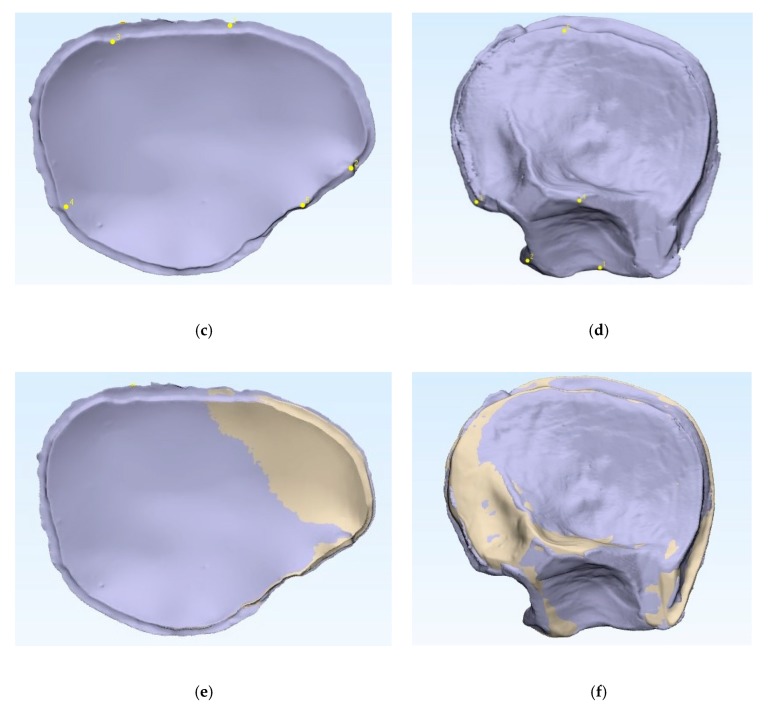
Comparison of the 3D printed templates (**a**,**b**, beige) with the patient-specific implants according to the n-point registration with five manually placed control points (**c**,**d**, purple), and the superimposition (**e**,**f**). Left: cranial template and PSI; right: temporo-orbital template and PSI.

To compare the fabricated PMMA PSIs’ accuracy with that of the 3D printed templates, the datasets were superimposed via the best-fit alignment method using a 3D analysis program (3-matic medical v. 13.0, Materialise, Leuven, Belgium). The accuracy of the PMMA PSIs was evaluated by superimposing the STL file data of the related template with the STL file data obtained from the CRD-PSIs (*n* = 10) or the TOD-PSIs (*n* = 10) test group. For accurate alignment, the datasets of the CRD- and TOD-PSIs were registered with the corresponding 3D printed templates. All registrations were achieved using the “align” feature. Therefore, five manually placed control points in the n-point registration and a global registration were performed (Figure 2). Following superimposition, the differences between the PSIs and the corresponding templates were compared with a maximally tolerated deviation of ± 2 mm. For each test group, the mean positive (the test group scan positioned in front of the reference scan) and negative deviation (the test group scan positioned behind the reference scan) were recorded. The measurements were visualized in a color map, including the scale of the deviations. Using an identical coordinate system between the datasets, the quantitative values of the deviations were automatically calculated using a 3D analysis program with respect to the root mean square (RMS) values. The RMS values describe the absolute values of the deviations between two datasets. This comparison of the two datasets comprising n-dimensional vector sets provides a measurable value of the similarity after optimal superimposition. The higher the RMS value is, the greater the deviation error between the two datasets will be.

### 2.6. Statistical Analyses

Descriptive statistics were used for the PSIs in both test groups. The data were represented as the mean difference, standard deviation, median difference, and the first and third quartile ranges. The Shapiro–Wilk test was applied to verify the normal distribution of the RMS values between the test groups. To determine a significant difference between the test groups, the Mann–Whitney U test was used. All parameters were measured in millimeters (mm). The level of statistical significance was set at α < 0.05. All statistical analyses were performed using R statistical software (R Core Team 3.4.1, The R Foundation for Statistical Computing, Vienna, Austria).

## 3. Results

**Figure 3:**
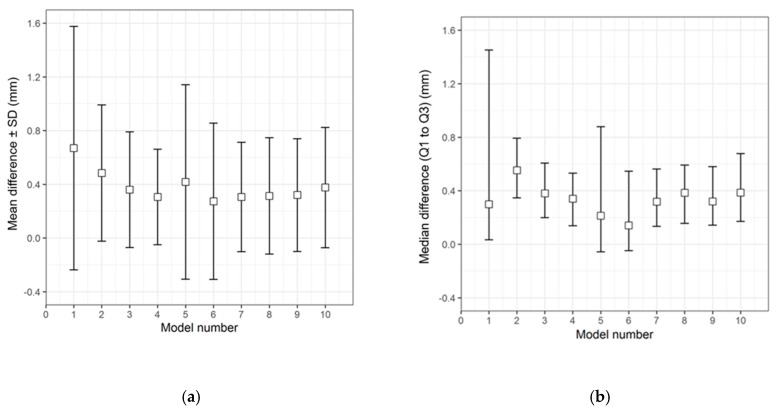
Descriptive data distribution illustrating the difference between the CRD-PSIs (models 1 to 10) and the CRD 3D printed template. (**a**) Mean difference ± SD (mm); (**b**) Median difference (Q1 to Q3) (mm).

**Figure 4:**
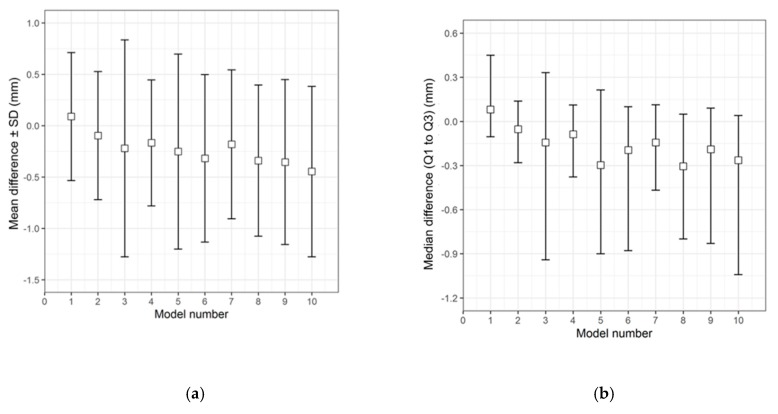
Descriptive data distribution illustrating the difference between the TOD-PSIs (models 1 to 10) and the TOD 3D printed template. (**a**) Mean difference ± SD (mm); (**b**) Median difference (Q1 to Q3) (mm).

The qualitative (descriptive) data distribution for the differences in the test groups, when compared with the corresponding 3D printed templates, is visualized in the following graphs (i.e., the CRD-PSIs with the CRD 3D printed template (Figure 3), and the TOD-PSIs with the TOD 3D printed template (Figure 4)).

The overall qualitative data distribution of the PSIs with the mean difference (± SD) and median difference (Q1 to Q3) were calculated for each test group. In the CRD-PSI test group, the comparative analyses revealed a mean difference ± SD of 0.383 (± 0.547) mm, while the median difference (Q1 to Q3) was 0.334 (0.122 to 0.722) mm. Meanwhile, the comparative analyses for the TOD-PSI test group revealed a mean difference ± SD of 0.228 (± 0.790) mm and a median difference (Q1 to Q3) of −0.159 (−0.662 to 0.165) mm (Table 2).

For the quantitative assessment of the accuracy of the PMMA PSIs with the corresponding 3D printed templates, the RMS values were analyzed. The highest RMS value or deviation error was observed in CRD-PSI 01 (1.128 mm), and CRD-PSI 04 had the lowest deviation error (0.469 mm). In the TOD-PSI test group, the highest and lowest deviation errors were observed in TOD-PSI 03 (1.079 mm) and TOD-PSIs 01/02 (0.630 mm), respectively (Table 3).

**Figure 5:**
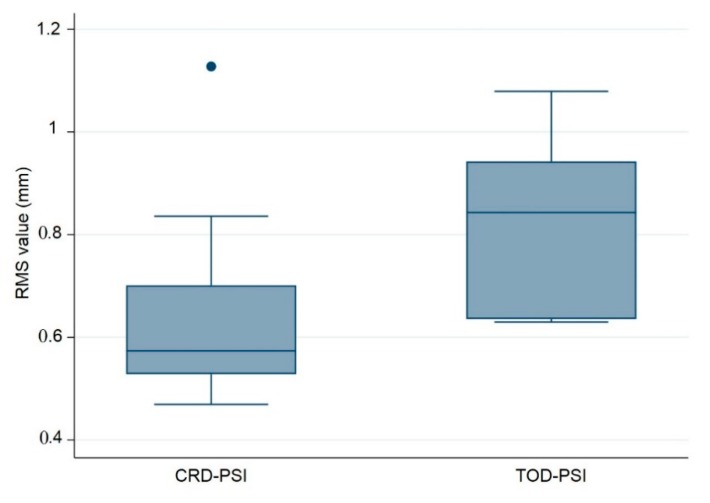
Box plot illustrating the accuracy comparison with respect to the root mean square (RMS) values (mm) between the polymethylmethacrylate (PMMA) CRD-PSI and TOD-PSI test groups (● describes the statistical outlier, CRD-PSI 01).

The following composite box plot graph represents the quantitative data distribution results of the RMS values for the ten (*n* = 10) PSIs within each test group (Figure 5).

Additionally, a quantitative assessment of accuracy with respect to the median RMS values revealed a statistically significant difference between the test groups, with a *p*-value of 0.028. This *p*-value is less than 0.05 and reflects the statistical significance between the CRD-PSIs and TOD-PSIs. The results of the RMS values show a median (Q1 to Q3) of 0.574 (0.528 to 0.701) mm for the CRD-PSIs, while the TOD-PSIs had a median (Q1 to Q3) RMS value of 0.843 (0.635 to 0.943) mm (Table 4).

**Figure 6:**
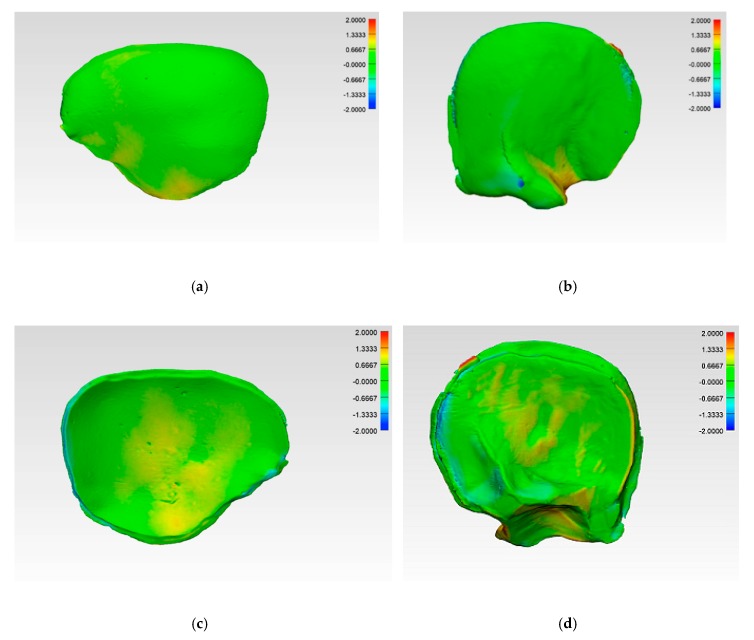
Color-coded deviation maps within each test group after applying the best-fit method and generating a 3D comparison to evaluate the accuracy. CRD-PSI: (**a**) squamous (outer) surface; (**c**) cerebral (inner) surface; TOD-PSI: (**b**) squamous (outer) surface; (**d**) cerebral (inner) surface.

The illustrations of the CRD-PSIs and TOD-PSIs for the deviation analysis are visualized in a heat map. The blue-colored areas of the heat map show a negative deviation while the red-colored areas show a positive deviation. The CRD-PSIs show a slightly positive deviation on the squamous (outer) surface of the temporal region of the PSIs, while a slightly negative deviation was observed on the cerebral (inner) surface at the antero- and posterolateral margins of the PSIs. By contrast, in the TOD-PSIs, a strong positive deviation was observed on the squamous (outer) surface at the infra-temporal region, and a negative deviation was observed on the cerebral (inner) surface at the posterolateral margin of the PSIs (Figure 6).

## 4. Discussion

Patient-specific treatment is becoming more and more popular in everyday medical practice and is also playing an increasingly important role in new diagnostic procedures, decision makings and treatments [18]. The demand for PSIs is constantly rising, especially in cranioplasty. In this field, the differences between the reconstruction of a skull defect with a manually manufactured implant and a PSI are clearly visible. Studies have quantified and assessed the accuracy of differences between machined and cast PMMA [19], the comparison between manual and automated techniques for cranioplasty plates [20], and the extent of conformity among pressed-titanium sheet implants on CRD contours [21]. Other studies examining 3D printed templates and PSIs often depict the accuracy or the evaluation of cosmetic outcomes based on CT scans, as well as patient questionnaires to obtain objective and subjective assessments [8,22]. Therefore, to investigate the outcome quality of a PSI in a more objective manner, the present study was conducted. The dimensional error assessment for the geometrical contours was based on a comparison of the surfaces of the PMMA PSIs with the surfaces of the virtually designed 3D printed templates. The authors aimed to establish a quantified criterion for the silicone mold-fabricated PMMA PSIs by analyzing the accuracy of the in-house fabrication procedure for two different craniectomy defect patterns.

The comparison between the 3D printed templates and the corresponding CRD- and TOD-PSIs revealed deviations. In the illustrated CRD case, the highest RMS value coincided with PSI 01 (1.128 mm), while the lowest RMS value was observed in PSI 04 (0.469 mm). In contrast, in the illustrated TOD case, PSI 03 had the highest RMS value (1.079 mm), while the lowest RMS values were observed in PSI 01 and 02 (0.630 mm) (Table 3). Since a lower RMS value indicates a decrease in error, the data reveal that in both the CRD and TOD test groups, an increase in accuracy is evident after the fifth model fabrication. This may be due to the fact that the PMMA silicone mold fabrication technique requires a certain level of dexterity and because the fabrication process has a learning curve. Wear issues can take effect in later stages, and an increase in errors due to mold tear was observed after the seventh model fabrication. The median RMS value of all CRD-PSIs compared to the corresponding CRD-template is 0.574 mm. Comparing the TOD-PSIs to the corresponding TOD-template, the median RMS value is 0.843 mm. The p-value of 0.028 (*p* < 0.05) shows a statistically significant deviation between the two test groups. However, this value did not indicate any clinically significant aberrance. The RMS values illustrate that, even after ten impressions (*n* = 10), the manufacturing method produces no clinically relevant deviations (Table 3). Overall, the results suggest that the manufacturing process described in this study is an exact and reproducible technique. The median RMS values for each of the two test groups did not exceed 1 mm, which is an acceptable accuracy for clinical routine in craniofacial reconstruction (Table 4) [23,24]. This finding represents a key indicator demonstrating that even after successive usage of silicone molds, the dimensional accuracy of PMMA PSIs meets a clinically acceptable accuracy level.

The comparison of the accuracy in the present study shows that CRD-PSIs had a comparatively lower deviation than the TOD-PSIs (Figure 4 and Figure 5, Table 4). A visual evaluation of the heat map reveals that the deviation distribution differed according to the shape of the PSI. In the heat map of the CRD-PSIs, a slightly positive deviation was observed on the central temporo-parietal region of the PSIs. The TOD-PSIs had a strong positive deviation in the infra-temporal region of the PSIs (an increase in material deposition in comparison to the 3D printed template). In both the CRD-PSIs and the TOD-PSIs, a negative deviation was observed on the cerebral surface at the antero- and posterolateral inner-ridge margins (Figure 6). This could have been caused by pressure-dependent displacement of the silicone mold during PSI fabrication. In the CRD-PSIs, which have a larger surface area, more negative deviations were found than in the TOD-PSIs, which have more positive deviation. The bigger and more complex the defect is, the thinner it is in the border area, and, depending on the curvature, this type of defect makes it more difficult to ensure a perfectly fitting PSI [25]. Hence, the location of the defect and the size and shape of the implants account for the different zones in which the deviations occur.

To reflect the manufacturing steps in which the PSIs deviations occur, each step must be analyzed. During the virtual planning phase of the template, 3D printing of the template, scanning of the template, fabrication of the mold, manufacturing of the PMMA implant, scanning of the PMMA implant, and the analyzing process there are many potential sources of error [26].

The resolution of the CT data, upon which the entire virtual planning of the template (as well as the manufacturing and comparison processes) are based, is an important factor for the outcome [27]. A slice separation of 1 mm should lead to accurate results for bone edges [25]. In the virtual planning phase, inaccuracies, such as those from automated, threshold-based reconstructions, may occur and the resulting template may no longer render the exact anatomy [28]. The type of 3D printing technology and material used are relevant factors, as they will determine the texture of the surface structure as well as the defects caused by shrinkage or warping. Printing errors can result in inaccuracies of the template. These deviations are then passed onto the new PSI during the molding process. To prevent these errors, careful attention was paid to the precise imaging techniques and accurate mold fabrication techniques that are the prerequisites for accurate PSIs.

The highest risk for PSIs deviations may occur at the beginning of the fabrication process. Currently, few materials for 3D printers that are certified for implantation are available. Consequently, non-biocompatible 3D printed templates must be transferred into a certified biocompatible material, such as bone cement [1,12,13,14,15,16,22,25,29]. Variations in the operator’s experience will influence the results, and there may be a learning curve for hand-mixed bone cement [30]. Besides the operator’s experience, the manufacturing time depends on several other factors, such as room temperature as well as the temperature and humidity of the monomer and polymer [17,30]. These external factors were kept consistent throughout the PSIs’ fabrication process so as not to influence the production time. The manufacturer’s instructions were followed exactly. After 30 seconds, the bone cement should be mixed to a homogeneous compound [17]. Several authors recommend vacuum mixing to enhance mechanical properties [31]. It should be noted that vacuum mixing leads to increased microporosities compared to hand mixing, which leads to the generation of macroporosities due to air entrapment in the material. These macroporosities, however, were minimized by decreasing the number of strokes and reducing the speed of hand mixing [32]. Next, the bone cement can be applied into the mold. If this step takes too long, the cement will begin to harden and cannot be molded accordingly. Consequently, the implant can become too thick, or the fine structures in the border areas cannot be reached by the material, resulting in an insufficient fit [33]. This can also be caused by pressure-dependent displacement of the silicone mold during PSI fabrication [12,34]. An equally distributed and constant pressure on the mold is crucial for a reproducible manufacturing process. The two parts of the mold must fit exactly to ensure a precise implant. During the fabrication process of PMMA PSIs, variability was inevitable on account of the manufacturer pressing tightly on the silicone molds. To eliminate the possible biases caused by one operator, two different surgeons were responsible for each case in this study.

Another source of deviation in PMMA PSIs can be explained by the shrinkage that occurs after the setting and curing time for PMMA. As soon as the cement hardens (but before the cement is fully cured), the implant must be removed from the mold to cut off the surpluses. On the one hand, if the implant is removed too early, the shape may become deformed due to the cement’s softness [25]. On the other hand, if the implant remains in the mold too long, it becomes difficult to remove and trim. This high stress may lead to wear and tear of the mold, which can impact the accuracy of future reproductions. After ten impressions, the thin parts at the border area or the regions with some undercuts particularly showed slight signs of wear. The fine structures of the mold can start to tear, and small parts of the mold may break off, so the implants show some deviations in these zones. After the implants are removed from the mold, the surpluses must be trimmed by hand. This must be done carefully to avoid deformation of the PSIs. Usually, PMMA PSIs are slightly adjusted prior to intraoperative miniplate fixation, which can give them an advantage over other implant materials (e.g., alloys) [29]. All these different factors during the overall manufacturing process play important roles and may influence the accuracy of the fabricated PSIs.

Although this was a laboratory study and we did not assess the clinical impact of the fabricated PSIs in patients, the present work has introduced quantitative assessments of accuracy during the pre-surgical phase. Another limitation of this study was that we did not access craniectomy defects crossing the midline. The full range of possible errors that may result from digital technologies, such as optical scanners and 3D printers, as well as material properties, have not been considered. However, the assessment of these errors lies beyond the scope of this article. Considering this study’s results, these errors can only contribute to inaccuracies to a small, clinically negligible extent. In summary, all the above-mentioned sources of error predominantly lead to small deviations that are irrelevant to craniofacial reconstruction.

The workflow described in this study offers an accurate and straightforward method for performing anatomical reconstructions using PSIs [12,35]. The most striking advantages of this process are the reduction of operation time [18,36], the use of common materials widely available, and the possibility to reuse the silicon mold in case of a revision operation. With the increase of the use of biocompatible materials [37] and bioprinting, these indirect processes, such as the molding of objects, will eventually become redundant in the future. Most likely, PSIs will be routinely 3D printed directly from PMMA bone cement, any other biocompatible material that is certified for implantation, or even of new autologous bone built from patients’ cells. Until then, the described manufacturing process remains a sophisticated means of producing patient-specific PMMA implants.

## 5. Conclusions

The present study demonstrates that the described manufacturing process of molded patient-specific PMMA implants based on 3D printed templates is highly precise, with a less than 1 mm deviation evaluated in two different defect patterns. This reflects the results commonly reported in the literature, where the overall inaccuracies of pure 3D printed anatomical models are also less than 1 mm [23]. Thus, the PSIs are largely consistent with the 3D printed templates in terms of accuracy. These results are reflected by the median RMS values, which are 0.574 mm for CRD-PSIs and 0.843 mm for TOD-PSIs. In addition, the silicon mold can withstand at least ten impressions without leading to clinically significant accuracy losses, which is sufficient in the case of a revision operation.

This workflow has proven to provide a sophisticated solution and continues to serve its purpose in everyday healthcare. It offers an accurate method for producing patient-specific PMMA implants until new, more economical methods are established for routine use.

## Figures and Tables

**Table 1:** Cone-beam computed tomography settings.

Implant	Volume	kV	mA	Voxel
CRD-PSI	17 × 13.5 cm^3^	90	5.0	300 µm
TOD-PSI	10 × 10 cm^3^	90	5.0	180 µm

**Table 2:** Overall results (mm) of the CRD-PSIs and TOD-PSIs compared to the corresponding 3D printed template.

Implant	Mean Difference ± SD	Median Difference (Q1 to Q3)
CRD-PSIs	0.383 ± 0.547	0.334 (0.122 to 0.722)
TOD-PSIs	0.228 ± 0.790	−0.159 (−0.662 to 0.165)

**Table 3:** Quantitative evaluation of the CRD-PSIs and TOD-PSIs accuracy with respect to the root mean square (RMS) values (mm).

Implant	RMS (mm)	Implant	RMS (mm)
CRD-PSI 01	1.128	TOD-PSI 01	0.630
CRD-PSI 02	0.701	TOD-PSI 02	0.630
CRD-PSI 03	0.562	TOD-PSI 03	1.079
CRD-PSI 04	0.469	TOD-PSI 04	0.635
CRD-PSI 05	0.836	TOD-PSI 05	0.983
CRD-PSI 06	0.644	TOD-PSI 06	0.875
CRD-PSI 07	0.510	TOD-PSI 07	0.747
CRD-PSI 08	0.535	TOD-PSI 08	0.811
CRD-PSI 09	0.528	TOD-PSI 09	0.878
CRD-PSI 10	0.585	TOD-PSI 10	0.943

Left: CRD-PSIs vs. CRD 3D printed template; right: TOD-PSIs vs. TOD 3D printed template.

**Table 4:** Quantitative accuracy assessment for the median RMS values (mm) in the CRD-PSIs and TOD-PSIs.

Implant	Median (Q1 to Q3)	*p*-Value *
CRD-PSIs	0.574 (0.528 to 0.701)	0.028
TOD-PSIs	0.843 (0.635 to 0.943)	

* *p*-value calculated using Mann–Whitney U test; * *p* < 0.05 is considered statistically significant.

## Abbreviations

CAD	Computer-aided design
CBCT	Cone-beam computed tomography
CRD	Cranial defect
CT	Computer tomography
DICOM	Digital imaging and communications in medicine
FFF	Fused filament fabrication
HA	Hydroxyapatite
HU	Hounsfield units
MIMICS	Materialise interactive medical image control system
MM	Millimeters
PEEK	Polyetheretherketone
PLA	Polylactic acid
PMMA	Polymethylmethacrylate
PSI	Patient-specific implant
RMS	Root mean square
SD	Standard deviation
STL	Standard tessellation language
Ti	Titanium
TOD	Temporo-orbital defect
2D	Two-dimensional
3D	Three-dimensional
